# Progressive Smartphone Restriction Combined with Psychoeducational Guidance and Pre-Sleep Autonomic Regulation Improves Sleep Efficiency and Time-of-Day Cognitive Performance in Physically Active Students with Nomophobia: A Randomized Controlled Trial

**DOI:** 10.3390/life16020212

**Published:** 2026-01-28

**Authors:** Wiem Ben Alaya, Wissem Dhahbi, Mohamed Abdelkader Souissi, Nidhal Jebabli, Halil İbrahim Ceylan, Nagihan Burçak Ceylan, Raul Ioan Muntean, Nizar Souissi

**Affiliations:** 1 Physical Activity, Sport and Health Research Unit (UR18JS01), National Observatory of Sports, Tunis 1003, Tunisia; wiembenalaya12@gmail.com (W.B.A.); gaddoursouissi@yahoo.com (M.A.S.); n_souissi@yahoo.fr (N.S.); 2 High Institute of Sport and Physical Education of El Kef, University of Jendouba, El Kef 7100, Tunisia; 3 Research Unit: Sport Sciences, Health and Movement, High Institute of Sport and Physical Education of El Kef, University of Jendouba, El Kef 7100, Tunisia; wisem.dhahbi@gmail.com (W.D.); jnidhal@gmail.com (N.J.); 4 Training Department, Qatar Police Academy, Police College, Doha 7157, Qatar; 5 High Institute of Sport and Physical Education of Sfax, Sfax University, Sfax 3000, Tunisia; 6 Faculty of Sports Sciences, Atatürk University, Erzurum 25240, Türkiye; 7 Graduate Education Institute, Bayburt University, Bayburt 69000, Türkiye; burcaksehitoglu@gmail.com; 8 Faculty of Law and Social Sciences, University “1 Decembrie 1918” of Alba Iulia, 510009 Alba Iulia, Romania; 9 High Institute of Sport and Physical Education, KsarSaid, University of Manouba, Mannouba 2010, Tunisia

**Keywords:** actigraphy, autonomic nervous system, breathing exercises, chronobiology disorders, psychomotor performance, sleep quality, technology addiction, vagus nerve

## Abstract

**Aim**: This study compared the effects of standard evening smartphone restriction with an adapted intervention combining progressive restriction, psychoeducational guidance, and pre-sleep relaxation on sleep, psychological state, cognitive performance, and physical performance in physically active physical education students with moderate-to-high nomophobia. **Methods**: Thirty participants (age 21.9 ± 1.2 years; intermediate chronotype) completed a randomized controlled trial consisting of a 7-day baseline period, a 14-day intervention phase, and post-intervention assessments. The standard group (*n* = 15) implemented a 2-h pre-bedtime smartphone restriction combined with general sleep hygiene guidance. The adapted group (*n* = 15) followed a progressive restriction protocol (30→60→120 min) supplemented with psychoeducational guidance targeting smartphone-related anxiety and a nightly slow-paced breathing routine. Objective sleep parameters were quantified using wrist-worn actigraphy. Subjective sleep quality, pre-sleep anxiety, and stress were assessed using visual analog scales. Cognitive performance (psychomotor vigilance task and choice reaction time) and physical performance (vertical jumps and agility) were evaluated at both morning and afternoon time points. **Results**: The adapted intervention produced significantly greater improvements in sleep efficiency (time × group: F(1,28) = 6.84, *p* = 0.014, ηp^2^ = 0.20; d = 0.78) and sleep onset latency (F(1,28) = 5.97, *p* = 0.021, ηp^2^ = 0.18; d = 0.72) compared with standard restriction. Significant reductions were also observed in pre-sleep anxiety (F(1,28) = 7.12, *p* = 0.012, ηp^2^ = 0.20; d = 0.81) and stress (F(1,28) = 6.45, *p* = 0.017, ηp^2^ = 0.19; d = 0.74). Cognitive performance showed significant time × group × time-of-day interactions, with improvements during afternoon assessments in psychomotor vigilance (F(1,28) = 7.48, *p* = 0.011; d = 0.83) and choice reaction time (F(1,28) = 6.89, *p* = 0.014; d = 0.79) exclusively in the adapted group. Physical performance outcomes remained stable across interventions. **Conclusions**: Progressive smartphone restriction combined with psychoeducational strategies and pre-sleep relaxation yields clinically meaningful improvements in sleep continuity, psychological arousal, and afternoon cognitive performance, exceeding the benefits achieved through behavioral restriction alone.

## 1. Introduction

Sleep is a fundamental physiological process that supports physical recovery, cognitive functioning, emotional regulation, and performance optimization in physically active individuals [1]. In university students and athletes, insufficient or disrupted sleep impairs sustained attention, delays reaction times, elevates psychological distress, and compromises training adaptation [2,3]. Despite growing recognition of sleep’s critical role in health and performance, young adults frequently experience sleep difficulties during periods of concurrent academic and physical demands, establishing an urgent need for effective, accessible interventions targeting modifiable sleep-disrupting behaviors [4].

Excessive evening smartphone use has emerged as a significant contributor to sleep disturbances among young adults, with approximately 48.5% reporting direct smartphone interference with sleep processes and 83.6% of university students demonstrating poor sleep quality [5]. Bedtime smartphone engagement delays sleep onset, reduces sleep efficiency, and degrades subjective sleep quality through multiple physiological and psychological pathways [1,4,6]. While short-wavelength light emission suppresses melatonin secretion and delays circadian phase [7], accumulating evidence indicates that cognitive and emotional activation induced by smartphone content contributes substantially to sleep disruption independent of photobiological mechanisms. Social media interactions, fear of missing out experiences, anticipatory vigilance regarding notifications, and compulsive checking behaviors elevate pre-sleep cognitive arousal, thereby prolonging sleep onset latency and fragmenting sleep continuity [8]. Experimental investigations confirm that restricting in-bed smartphone use improves sleep quality through multiple interacting pathways, including reduced pre-sleep cognitive arousal, minimized light-induced circadian disruption, attenuated behavioral conditioning of bed-wakefulness associations, and prevention of sleep curtailment from extended device engagement [9]. The relative contribution of these mechanisms remains incompletely characterized, though accumulating evidence indicates that cognitive and emotional activation contribute substantially to sleep disruption independent of photobiological pathways [8,9].

These cognitive–emotional mechanisms demonstrate particular salience among individuals with elevated nomophobia, operationally defined as anxiety and discomfort arising from smartphone unavailability [10]. Although nomophobia’s status as a distinct clinical entity remains debated, with ongoing discussions regarding conceptual overlap with generalized anxiety, fear of missing out, and problematic technology use [11], it provides a useful heuristic for identifying individuals experiencing heightened distress during smartphone separation. Nomophobia affects approximately 66% of individuals globally, with prevalence reaching 68.6% in specific adult populations and elevated severity among university students [5]. Individuals exhibiting moderate-to-high nomophobia report difficulty disengaging from smartphones during evening hours, heightened pre-sleep worry, and pronounced discomfort during intentional disconnection, processes closely aligned with cognitive hyperarousal models of insomnia [12]. Recent mediation analyses indicate that nomophobia predicts poor sleep quality via pre-sleep arousal pathways, with cognitive arousal exerting stronger effects than somatic arousal [13]. This evidence aligns with contemporary insomnia frameworks that emphasize pre-sleep cognitive and physiological hyperarousal as core perpetuating factors maintaining sleep initiation difficulties.

Experimental evidence shows that the mere presence of a smartphone has little to no effect on cognitive performance under passive or task-based laboratory conditions. A recent four-level meta-analysis synthesizing 166 effect sizes from 53 samples (33 studies; *n* = 4368) reported a non-significant overall effect of smartphone proximity on cognitive performance (d = −0.02, 95% CI: −0.06 to 0.01) [14]. These findings highlight that interventions targeting active and arousal-inducing smartphone behaviors, particularly during the pre-sleep period, when anxiety, vigilance, and compulsive checking tend to peak [8], offer a more ecologically valid and theoretically grounded approach than passive-exposure paradigms. By focusing on intentional disengagement and pre-sleep arousal reduction, the present design addresses mechanisms central to sleep disruption in nomophobic populations, mechanisms that are unlikely to be detected by simple mere-presence protocols.

The timing of performance assessment represents a critical methodological consideration when evaluating sleep-focused interventions. Cognitive performance exhibits pronounced diurnal variation driven by circadian rhythms and homeostatic sleep–wake regulation [3,15]. Sustained attention and reaction time are particularly vulnerable during periods when homeostatic sleep pressure accumulates against declining endogenous circadian arousal [15]. In individuals with intermediate chronotypes assessed during typical academic schedules, this vulnerability manifests prominently during post-lunch afternoon hours (approximately 13:00–16:00 h), though the temporal window of maximal impairment varies substantially with chronotype, prior sleep history, and circadian phase [3,15]. This vulnerability arises because sleep restriction elevates adenosine accumulation and disrupts prefrontal cortical activation, processes that manifest most prominently when circadian arousal signals decline during the post-lunch afternoon hours [3,16]. In physically active young adults, afternoon cognitive impairment following insufficient sleep may compromise academic performance and training adaptation, underscoring the functional relevance of time-specific assessments. Sleep deprivation selectively impairs late-day cognitive function by disrupting homeostatic-circadian interactions, suggesting that interventions that improve sleep architecture may yield time-of-day-dependent benefits obscured by single-time-point assessments. Similarly, neuromuscular and anaerobic physical performance frequently demonstrates afternoon superiority over morning assessments, reflecting circadian modulation of core body temperature, neuromuscular efficiency, and metabolic processes [17,18]. This pattern exhibits moderation by training status, time-of-day adaptation history, chronotype, and environmental conditions, with trained athletes demonstrating attenuated diurnal amplitude when training consistently at non-optimal circadian phases [17]. These temporal dynamics underscore the need for multiple daily assessments when examining the functional outcomes of sleep interventions.

Current recommendations for mitigating smartphone-related sleep disturbances emphasize behavioral restriction strategies, typically advising complete device abstention during the one to two hours preceding habitual bedtime [1]. While restriction protocols may benefit certain individuals, meta-analytic evidence indicates modest effect sizes for screen time reduction on sleep quality when implemented without psychological support [19]. Restriction-only approaches may demonstrate limited efficacy in nomophobic populations. Theoretical models suggest that abrupt disconnection could paradoxically elevate anticipatory anxiety and cognitive arousal, potentially undermining adherence and therapeutic benefit [8]. However, experimental evidence directly demonstrating systematic sleep deterioration following smartphone restriction in nomophobic individuals remains limited, rendering this hypothesis plausible but incompletely validated. Psychoeducational strategies grounded in cognitive–behavioral principles address maladaptive beliefs regarding smartphone unavailability and promote adaptive coping responses [12]. Complementary relaxation techniques, including slow-paced diaphragmatic breathing, enhance vagally mediated heart rate variability and improve autonomic regulation during practice sessions [20,21]. Evidence for breathing technique effects on objective sleep parameters assessed via polysomnography or actigraphy remains limited and inconsistent [22], with the most robust effects observed for subjective sleep quality and pre-sleep psychological tension rather than sleep architecture [23]. However, these subjective improvements may translate into functional benefits when combined with behavioral restriction and psychoeducation. Recent randomized controlled trials of multicomponent interventions that integrate behavioral modification with psychological support have demonstrated superior outcomes compared with restriction alone, particularly when targeting pre-sleep arousal mechanisms [24,25]. However, the combined effects of progressive restriction, psychoeducation, and pre-sleep autonomic regulation on sleep and time-of-day-dependent cognitive and physical performance remain insufficiently characterized in physically active populations with elevated nomophobia. Sleep deprivation selectively impairs late-day cognitive function by disrupting homeostatic-circadian interactions, suggesting that interventions that improve sleep architecture may yield time-of-day-dependent benefits obscured by single-time-point assessments [3].

Randomized trials integrating behavioral restriction with psychoeducational support have demonstrated superior outcomes compared with restriction alone [24,25]. However, multicomponent interventions combining progressive restriction protocols, cognitive–emotional psychoeducation targeting smartphone-related anxiety, and structured pre-sleep autonomic regulation remain underexplored, particularly regarding time-of-day-dependent cognitive and physical performance outcomes in physically active populations with elevated nomophobia. This study compared standard smartphone restriction with an adapted intervention integrating progressive restriction protocols, psychoeducational guidance targeting smartphone-related anxiety, and structured pre-sleep relaxation incorporating slow-paced breathing techniques. We hypothesized that the adapted intervention would produce greater improvements in sleep efficiency and sleep onset latency, enhance subjective sleep quality, and reduce pre-sleep anxiety and stress. Given the modest anticipated magnitude of sleep improvement (~30–60 min total sleep time increase) and the 14-day intervention duration, we expected cognitive performance benefits to emerge more readily than physical performance changes, as cognitive restoration occurs rapidly following sleep improvement [2], whereas neuromuscular adaptations typically require extended training-recovery cycles [26]. We further hypothesized that cognitive benefits would manifest preferentially during afternoon assessments, when homeostatic sleep pressure and cognitive vulnerability peak in this population [3,15].

## 2. Methods

### 2.1. Participants

Thirty physically active physical education students with moderate to high nomophobia volunteered for this randomized controlled trial. All participants were enrolled in university-level physical education and sport sciences programs and maintained regular structured physical activity (≥3 training sessions·week^−1^ for ≥2 years) as part of academic curriculum and extracurricular training. Nomophobia severity was assessed during screening using the Nomophobia Questionnaire (NMP-Q), a validated 20-item self-report instrument quantifying anxiety and discomfort associated with smartphone unavailability [27]. Only individuals classified as moderate (NMP-Q score 60–99) or high (≥100) were eligible [27]. The final cohort (*n* = 30) comprised individuals distributed across moderate (*n* = 24; 80%) and high (*n* = 6; 20%) severity categories, with baseline NMP-Q scores ranging from 68 to 103 (overall mean: 83.87 ± 7.62). Chronotype was assessed using the Morningness–Eveningness Questionnaire (MEQ) [27]. Only intermediate chronotypes (MEQ scores 42–58) were retained to minimize circadian heterogeneity arising from extreme morningness or eveningness preferences [18]. While substantial individual variation in circadian phase persists within this intermediate range (phase angle differences up to 2–3 h), restricting enrollment to MEQ 42–58 substantially reduces the confounding influence of extreme circadian phenotypes on sleep timing, duration, and time-of-day performance assessments [28]. The cohort comprised 19 men (63.3%) and 11 women (36.7%). Baseline anthropometric and psychological characteristics were age 21.9 ± 1.2 years, height 175.8 ± 4.2 cm, body mass 70.8 ± 3.2 kg, and NMP-Q score 83.87 ± 7.62. Between-group comparisons confirmed no baseline differences in age, anthropometry, or nomophobia severity (all *p* > 0.05) (Table 1).

Exclusion criteria encompassed diagnosed sleep disorders (e.g., insomnia, obstructive sleep apnea), neurological or psychiatric conditions, current use of sleep medication or psychoactive substances, recent musculoskeletal injury (<3 months), and any medical condition potentially interfering with sleep architecture, cognitive function, or physical performance. Participants maintained habitual training routines, dietary patterns, and sleep schedules throughout the study and refrained from initiating novel sleep- or recovery-oriented interventions. Sample size was determined a priori using G*Power software (version 3.1.9.2) [29]. Assuming α = 0.05, power (1 − β) = 0.80, and an expected effect size of f = 0.40, the analysis indicated that 30 participants would provide adequate statistical power to detect meaningful group × time interactions. The f = 0.40 estimate was derived from Exelmans and Van den Bulck [30], who reported Cohen’s d values of 0.51–0.72 for changes in sleep efficiency and onset latency following bedtime smartphone restriction in adults, corresponding to f ≈ 0.36–0.50 when converted to mixed-model ANOVA frameworks [29]. This conservative estimate balances detection sensitivity with feasibility constraints in specialized populations (intermediate chronotype, moderate-to-high nomophobia, physically active) where recruitment intensity exceeds that of general undergraduate samples. All participants provided written informed consent. The study protocol received approval from the Ethics Committee of the Faculty of Medicine of Sfax (reference 49/2024, date: 10 July 2024) and was conducted in accordance with the Declaration of Helsinki.

### 2.2. Experimental Design

**Figure 1:**
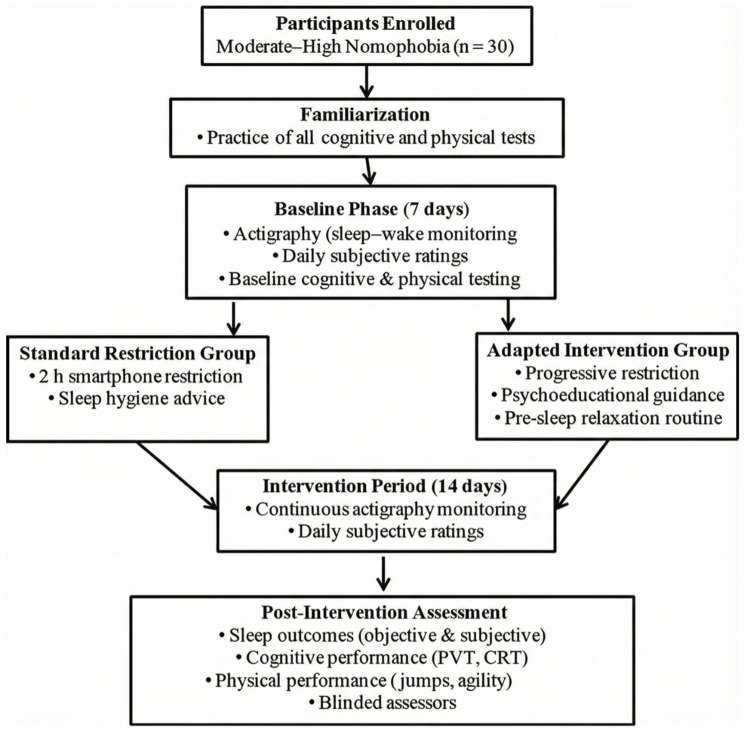
Experimental Design and Procedural Flowchart of the Randomized Controlled Trial Comparing Standard Smartphone Restriction with Adapted Smartphone Intervention.

A parallel-group randomized controlled design was implemented, comprising familiarization, baseline assessment, intervention, and post-intervention testing phases (Figure 1). Participants completed one familiarization session prior to baseline to attenuate learning effects on cognitive and physical performance measures [31]; familiarization data were excluded from analysis. During the 7-day baseline phase, habitual sleep–wake patterns were monitored continuously via wrist actigraphy, and daily subjective ratings (sleep quality, pre-sleep anxiety, pre-sleep stress) were recorded. Baseline cognitive and physical performance batteries were administered during the final 24 h of this phase.

Following baseline assessment, participants were stratified by nomophobia severity (moderate vs. high) and randomly allocated to intervention groups using a computer-generated random sequence (1:1 ratio) prepared by an independent statistician. Allocation concealment was maintained via sequentially numbered, opaque, sealed envelopes opened only after baseline assessments were finalized. Group assignments were revealed to participants immediately prior to intervention commencement but remained concealed from assessors conducting cognitive and physical performance evaluations throughout the study. The 14-day intervention phase maintained continuous actigraphy and daily subjective assessments. Post-intervention testing was conducted 24–48 h after completion of the intervention, replicating baseline procedures and temporal sequencing. Cognitive and physical assessments were conducted at two time points per day (morning: 09:00 h; afternoon: same clock time across sessions) at both baseline and post-intervention to capture potential time-of-day interactions, consistent with documented circadian modulation of neuromuscular and cognitive function [17]. The test order was standardized and identical for all participants: psychomotor vigilance task, choice reaction time task, squat jump, countermovement jump, and agility test. A 5-min passive recovery interval separated each test. This interval duration was selected based on evidence from validated sleep–performance and psychophysiological paradigms demonstrating that 3–5 min of passive rest provides sufficient physiological restitution for brief cognitive tasks, with minimal carryover when protocols are standardized [16]. Similarly, sport performance studies indicate that short explosive physical tests permit adequate neuromuscular recovery within 3–5 min when not performed to exhaustion [17]. Although complete elimination of residual fatigue cannot be guaranteed, the combination of fixed test sequencing, uniform recovery intervals, and brief task durations minimizes systematic bias attributable to task interference. Assessors conducting cognitive and physical evaluations were blinded to group allocation to reduce measurement bias. Participants abstained from caffeine and strenuous exercise for 24 h prior to each testing session and maintained a consistent food intake across corresponding time points to control for nutritional variability.

### 2.3. Intervention Protocols

The intervention design intentionally incorporated multicomponent elements (progressive restriction, psychoeducation, pre-sleep relaxation) exclusively in the adapted group to isolate the incremental benefit of these strategies beyond standard behavioral restriction. This asymmetry introduces potential expectancy effects, wherein participants receiving additional psychoeducational content and structured relaxation may anticipate superior outcomes, thereby influencing subjective ratings (sleep quality, anxiety, stress) independent of physiological mechanisms. However, convergence between objective actigraphic indices (sleep efficiency, sleep onset latency) and subjective ratings mitigates pure expectancy interpretations, as actigraphy quantifies sleep continuity independently of participant belief systems [32,33]. Furthermore, cognitive performance outcomes (psychomotor vigilance, choice reaction time) demonstrate minimal susceptibility to expectancy effects, particularly when assessed via objective computerized tasks with millisecond-precision measurement [31]. The asymmetric design thus permits evaluation of whether multicomponent strategies confer benefits exceeding those achievable through restriction alone, a clinically relevant comparison informing intervention optimization [24,25].

#### 2.3.1. Standard Smartphone Restriction

Participants abstained from all smartphone use during the 2-h period preceding habitual bedtime, defined as complete device non-interaction (no screen activation, notification checking, or passive device handling). Devices were powered off or placed in airplane mode and stored outside the bedroom overnight. Emergency contact accessibility was maintained via a designated landline or secondary basic-function phone (non-internet-capable) available to all participants. Notifications were disabled during restriction periods; no calls, messages, or application use were permitted. Concurrent sleep hygiene recommendations included maintaining consistent sleep–wake schedules, avoiding caffeine after mid-afternoon, and minimizing evening exposure to high-intensity or blue-enriched light [34].

#### 2.3.2. Adapted Smartphone Intervention

The adapted protocol integrated progressive behavioral restriction with psychoeducational guidance and structured pre-sleep psychophysiological regulation, designed to mitigate smartphone-related anticipatory anxiety and pre-sleep arousal [12]. Progressive restriction followed identical non-use parameters (complete device non-interaction, powered-off/airplane mode, bedroom exclusion, emergency-access provisions) but was implemented incrementally: 30 min pre-bedtime restriction (days 1–3), extended to 60 min (days 4–7), and increased to 120 min (days 8–14), equalizing final-week restriction with the standard group while attenuating withdrawal-related discomfort. Psychoeducational guidance incorporated cognitive–behavioral insomnia frameworks targeting anticipatory anxiety, cognitive hyperarousal, and emotional dependence on digital connectivity [12]. Content-normalized transient discomfort during intentional disconnection promoted cognitive reframing of smartphone unavailability as a recovery-oriented behavior and provided coping strategies for managing urges to check. This approach addressed established mechanisms whereby fear of missing out and pre-sleep cognitive arousal contribute to delayed sleep onset and reduced sleep quality [8]. The pre-sleep relaxation routine consisted of a standardized 10–12-min protocol performed nightly, combining slow-paced diaphragmatic breathing (5–6 breaths·min^−1^ with prolonged exhalation) and attentional regulation. Slow-paced breathing enhances vagal regulation and improves stress-related physiological markers [20], and paced-breathing techniques have been shown to be effective in addressing insomnia-related mechanisms [22]. Adherence was verified via daily self-report logs. Participants completed daily adherence logs documenting restriction start times, duration, and any protocol deviations. While objective smartphone-use monitoring (e.g., application-based screen-time tracking) would enhance adherence verification, logistical constraints and heterogeneity in participant device operating systems precluded standardized implementation during the study period.

### 2.4. Data Collection

#### 2.4.1. Objective Sleep Parameters

Sleep was quantified using wrist-worn triaxial accelerometers (ActiGraph wGT3X-BT; ActiGraph LLC, Pensacola, FL, USA) positioned on the non-dominant wrist continuously throughout baseline and intervention phases, except during water-based activities. Actigraphy provides a valid field-based assessment of sleep–wake patterns and sleep continuity with established concordance to polysomnography [32,33]. Data were processed using manufacturer-recommended algorithms and sleep-scoring procedures. Extracted variables included sleep efficiency (%), sleep onset latency (min), total sleep time (min), and wake after sleep onset (min). Bedtime and wake times were cross-verified using participant-maintained sleep diaries. To reduce night-to-night variability, sleep indices were averaged across the final three nights of the baseline and intervention periods. This aggregation strategy reflects standard actigraphic practice and is commonly used to obtain stable and reliable phase-level estimates by attenuating first-night effects and transient nightly fluctuations [35]. While averaging across three nights is widely accepted as a minimum for stabilizing actigraphic sleep parameters, this approach does not fully capture early intervention adaptation dynamics or high inter-night variability, particularly during the initial phase of behavior change.

#### 2.4.2. Subjective Sleep, Anxiety, and Stress

Subjective sleep quality was assessed each morning using a visual analog scale (VAS) ranging from 0 (very poor sleep) to 10 (excellent sleep). Pre-sleep anxiety and stress were evaluated each evening separately immediately before bedtime using an analogous VAS (0–10), with higher scores indicating greater perceived anxiety or stress. VAS methods demonstrate sensitivity to short-term changes in subjective sleep and affective states in repeated-measures paradigms [36].

#### 2.4.3. Cognitive Performance Assessment

Cognitive testing was conducted using PsyToolkit, an open-source platform that enables controlled stimulus presentation and response recording [37]. Sustained attention was assessed using a 5-min psychomotor vigilance task (PVT) requiring rapid response to visual stimuli presented at random inter-stimulus intervals (2–10 s). Primary outcomes were reaction time [20] and lapses (reaction time ≥ 500 ms), consistent with established PVT conventions and demonstrated sensitivity to sleep-related impairment [16,31]. Choice reaction time (CRT) was evaluated using a four-choice visual discrimination paradigm. Reaction time [20] and response accuracy (%) were recorded to quantify decision speed and evaluate potential speed–accuracy trade-offs [3].

#### 2.4.4. Physical Performance Assessment

Agility was quantified using a standardized change-of-direction test monitored with Witty photoelectric timing gates (Microgate, Bolzano, Italy). Participants initiated from a standardized stance (front foot 0.5 m posterior to the first gate) and, upon auditory signal, completed a predefined course emphasizing acceleration, deceleration, and rapid directional changes. Two maximal trials were performed with 2-min passive recovery; the fastest time(s) were retained. Vertical jump performance was assessed using the OptoJump optical measurement system (version 1.12.1; Microgate, Bolzano, Italy). Squat jump [26] required maintaining a static semi-squat position (~90° knee flexion) for 2 s to eliminate countermovement before executing a maximal vertical jump with hands positioned on hips. Countermovement jump (CMJ) involved a maximal jump with self-selected countermovement depth and hands on hips. For each jump modality, three trials were performed with 60-s passive recovery; maximal jump height (cm) was retained. OptoJump estimates jump height from flight time and has demonstrated established validity in athletic populations.

### 2.5. Statistical Analysis

All analyses were conducted using STATISTICA software (version 12; StatSoft, Maisons-Alfort, France). Linear mixed-effects models (LMMs) were applied to accommodate repeated measures and inter-individual variability, with participants specified as random intercepts. For objective sleep variables and subjective sleep quality, the fixed effects model included time (baseline vs. post-intervention), group (adapted vs. standard), and the time × group interaction. Anxiety and stress (assessed once daily pre-sleep) employed identical fixed-effect structures. Cognitive outcomes (PVT reaction time, lapses, CRT reaction time, CRT accuracy) and physical outcomes (SJ, CMJ, agility) were incorporated, along with time, group, time-of-day (morning vs. afternoon), and relevant interaction terms. Daily VAS ratings (sleep quality, pre-sleep anxiety, pre-sleep stress) were collected throughout baseline and intervention phases to capture day-to-day variability. For statistical analysis, individual daily ratings were averaged across the final three days of each phase (baseline days 5–7; intervention days 12–14) to derive stable phase-specific estimates while minimizing night-to-night fluctuation. These phase-averaged values were then entered into linear mixed-effects models with time (baseline vs. post-intervention) and group (adapted vs. standard) as fixed effects, and participant as a random intercept. This approach balances sensitivity to intervention-related change with robustness against transient daily variability [38]. When significant interactions emerged, Bonferroni-adjusted post hoc comparisons were performed. Statistical significance was set at α = 0.05. Effect sizes were quantified using Cohen’s d for pairwise comparisons and partial eta-squared (ηp^2^) for omnibus ANOVA effects. In accordance with discipline-specific guidelines derived from a large meta-analysis in rehabilitation research, Cohen’s d values were interpreted as small = 0.10, medium = 0.40, large = 0.80 [39]. Partial eta-squared values were interpreted using conventional ANOVA benchmarks (small = 0.01, medium = 0.06, large = 0.14) [40]. Model assumptions were verified through residual diagnostics. Results are reported as estimated marginal means with 95% confidence intervals where appropriate.

## 3. Results

### 3.1. Baseline Equivalence for Primary Outcome Variables

Baseline descriptive statistics for primary outcome variables are presented in Table 2. Independent-samples *t*-tests confirmed no statistically significant between-group differences for any sleep parameter (sleep efficiency: *p* = 0.29; sleep onset latency: *p* = 0.14; total sleep time: *p* = 0.17), subjective ratings (sleep quality: *p* = 0.48; pre-sleep anxiety: *p* = 0.58; stress: *p* = 0.52), or cognitive performance indices (PVT reaction time: *p* = 0.64; CRT reaction time: *p* = 0.60). These findings establish baseline equivalence, supporting the validity of subsequent intervention comparisons.

### 3.2. Objective Sleep Parameters

Linear mixed-effects modeling revealed a statistically significant time × group interaction for sleep efficiency (F(1,28) = 6.84, *p* = 0.014, ηp^2^ = 0.20), indicating differential intervention effects between groups. Bonferroni-adjusted post hoc comparisons demonstrated that the adapted smartphone intervention increased sleep efficiency from baseline to post-intervention (*p* < 0.01, d = 0.78), indicating a large effect. Conversely, the standard restriction group exhibited negligible change (*p* > 0.05, d = 0.22). Sleep onset latency exhibited a parallel pattern, with a significant time × group interaction (F(1,28) = 5.97, *p* = 0.021, ηp^2^ = 0.18). Participants assigned to the adapted intervention demonstrated a significant reduction in sleep onset latency following the intervention period (*p* < 0.05, d = 0.72), whereas no statistically significant change was observed in the standard restriction group (*p* > 0.05, d = 0.19).

For total sleep time, the time × group interaction did not attain statistical significance (F(1,28) = 1.12, *p* = 0.30, ηp^2^ = 0.04). However, a significant main effect of time was observed (F(1,28) = 4.21, *p* = 0.049, ηp^2^ = 0.13), reflecting a modest increase in total sleep time across both intervention groups. The magnitude of this change was small to moderate (d ≈ 0.35). Wake after sleep onset yielded no significant time × group interaction (F(1,28) = 0.88, *p* = 0.36, ηp^2^ = 0.03), with neither group exhibiting substantive pre-post alterations (d < 0.30) (Table 3). Collectively, these findings indicate that the adapted smartphone intervention produced specific and clinically relevant improvements in sleep efficiency and sleep initiation, whereas changes in sleep duration and nocturnal awakenings were modest and attributable to general time-related effects rather than differential intervention impact.

### 3.3. Subjective Sleep Quality

**Figure 2:**
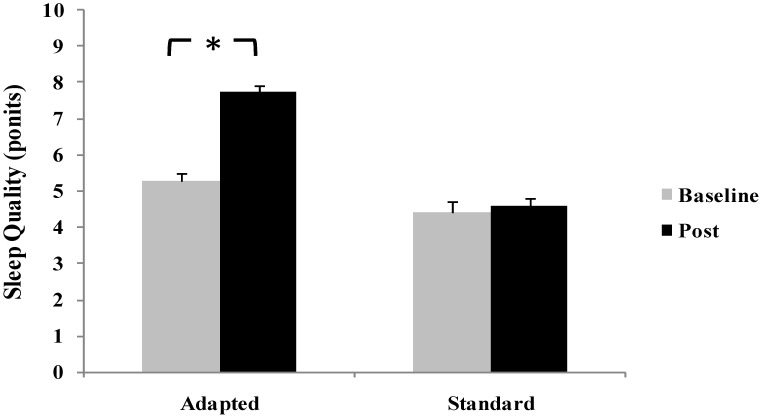
Changes in Subjective Sleep Quality Scores from Baseline to Post-Intervention in Standard Smartphone Restriction and Adapted Smartphone Intervention Groups. * *p* < 0.05.

Analysis of subjective sleep quality revealed a significant time × group interaction (F(1,28) = 6.31, *p* = 0.018, ηp^2^ = 0.18). Post hoc testing indicated that participants in the adapted intervention group reported a marked improvement in perceived sleep quality from baseline to post-intervention (*p* < 0.01, d = 0.76), corresponding to a large effect size. In contrast, the standard restriction group demonstrated minimal change that failed to reach statistical significance (*p* > 0.05, d = 0.23) (Figure 2).

### 3.4. Pre-Sleep Anxiety and Stress

**Figure 3:**
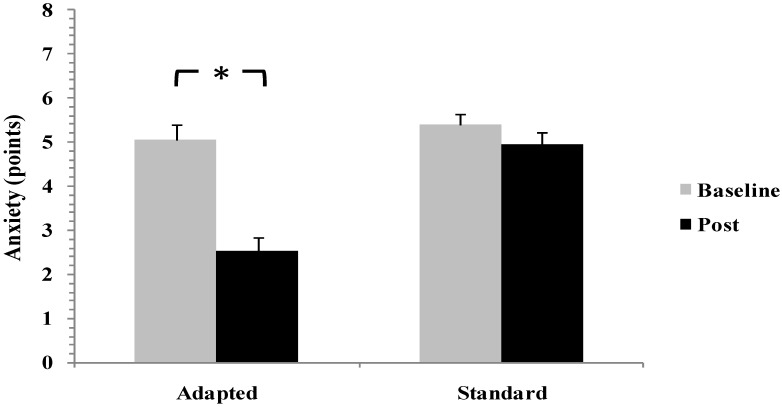
Changes in Pre-Sleep Anxiety Scores from Baseline to Post-Intervention in Standard Smartphone Restriction and Adapted Smartphone Intervention Groups. * *p* < 0.05.

For pre-sleep anxiety, linear mixed-effects modeling identified a significant time × group interaction (F(1,28) = 7.12, *p* = 0.012, ηp^2^ = 0.20), indicating superior reduction in the adapted intervention condition. Anxiety scores decreased significantly in the adapted group (*p* < 0.01, d = 0.81), representing a large effect, whereas changes in the standard group were negligible and non-significant (d = 0.24) (Figure 3).

**Figure 4:**
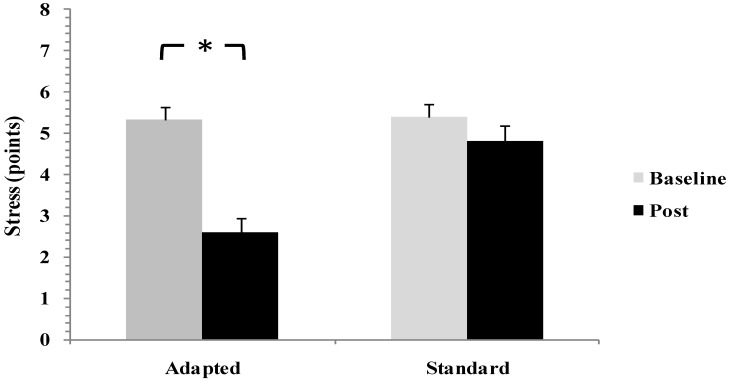
Changes in Pre-Sleep Stress Scores from Baseline to Post-Intervention in Standard Smartphone Restriction and Adapted Smartphone Intervention Groups. * *p* < 0.05.

Pre-sleep stress exhibited a comparable pattern, with a significant time × group interaction (F(1,28) = 6.45, *p* = 0.017, ηp^2^ = 0.19). Stress levels decreased significantly following the adapted intervention (*p* < 0.05, d = 0.74), corresponding to a large effect size. No meaningful changes were observed in the standard restriction group (d = 0.21) (Figure 4). These results demonstrate that the adapted smartphone intervention effectively attenuated pre-sleep psychological arousal, whereas simple smartphone restriction alone proved insufficient to elicit comparable reductions.

### 3.5. Cognitive Performance

For sustained attention, linear mixed-effects modeling revealed a significant time × group × time-of-day interaction for psychomotor vigilance task reaction time (F(1,28) = 7.48, *p* = 0.011, ηp^2^ = 0.21). Decomposition of this three-way interaction via Bonferroni-adjusted comparisons demonstrated that participants in the adapted intervention group exhibited a significant reduction in reaction time during afternoon assessments (*p* < 0.01, d = 0.83), corresponding to a large effect size. No significant changes were detected during morning sessions or in the standard intervention group across either time point.

The frequency of vigilance lapses (reaction time ≥ 500 ms) followed an analogous pattern, yielding a significant three-way interaction (F(1,28) = 6.62, *p* = 0.016, ηp^2^ = 0.19). Lapses decreased significantly in the adapted group during afternoon testing (*p* < 0.05, d = 0.71), whereas no alterations were observed in the standard group. Choice reaction time demonstrated a significant time × group × time-of-day interaction (F(1,28) = 6.89, *p* = 0.014, ηp^2^ = 0.20). The adapted intervention group exhibited faster decision-making performance during afternoon assessments (*p* < 0.01, d = 0.79), while performance remained stable in the morning and across all time points in the standard group.

For choice reaction time accuracy, linear mixed-effects modeling revealed no significant main effects of group, time, or time of day, nor any significant interaction effects (all *p* > 0.20). Response accuracy remained consistently elevated across all experimental conditions (mean ≈ 95–96%), indicating that improvements in reaction speed observed in the adapted intervention group were not achieved at the expense of decisional accuracy (Table 4).

### 3.6. Physical Performance

Linear mixed-effects modeling revealed no significant time × group interactions for squat jump, countermovement jump, or agility performance (all *p* > 0.05), with uniformly small effect sizes (d < 0.30). Significant main effects of time of day emerged for squat jump (F(1,28) = 5.62, *p* = 0.025, ηp^2^ = 0.17), countermovement jump (F(1,28) = 6.94, *p* = 0.013, ηp^2^ = 0.20), and agility (F(1,28) = 7.21, *p* = 0.012, ηp^2^ = 0.21), with superior afternoon performance (Table 5).

## 4. Discussion

This study compared standard evening smartphone restriction with an adapted intervention incorporating progressive restriction, psychoeducational guidance, and pre-sleep relaxation in physically active physical education students with moderate-to-high nomophobia. The adapted intervention produced large improvements in sleep efficiency (d = 0.78), sleep onset latency (d = 0.72), pre-sleep anxiety (d = 0.81), and stress (d = 0.74), alongside afternoon-specific enhancements in psychomotor vigilance (d = 0.83) and choice reaction time (d = 0.79). Physical performance remained stable beyond expected diurnal variations. These findings support a mechanistic framework in which interventions targeting pre-sleep arousal pathways produce meaningful improvements in sleep architecture with downstream cognitive benefits in nomophobic populations. The superior sleep efficiency and reduced sleep onset latency observed in the adapted group are consistent with theoretical models emphasizing cognitive–emotional activation pathways [8,12]. However, the study design did not independently manipulate or control light exposure (wavelength, intensity, duration), precluding comparative evaluation of photobiological versus psychological mechanisms. Both pathways likely contribute interactively to smartphone-related sleep disruption [7], with their relative importance remaining empirically unresolved in the present investigation. Definitive mechanistic attribution requires direct mediator measurement (validated pre-sleep arousal scales, autonomic indices), which was not incorporated in this design [41]. Emerging evidence indicates that social vigilance, fear of missing out, and anticipatory checking behaviors delay sleep onset through sustained pre-sleep cognitive arousal [8]. The psychoeducational component explicitly addressed these mechanisms through cognitive–behavioral insomnia frameworks, emphasizing maladaptive beliefs and pre-sleep worry as central drivers of sleep initiation difficulty [12]. By normalizing transient discomfort and reframing smartphone unavailability as recovery-oriented behavior, the intervention likely reduced threat appraisals associated with restriction. This interpretation aligns with experimental evidence that reducing in-bed smartphone use improves sleep, partly by decreasing pre-sleep cognitive arousal [9], supporting the integration of psychoeducational components beyond simple behavioral prohibition.

Nomophobia affects approximately 66% of individuals globally, with elevated prevalence among university students and young adults [42]. Recent cross-sectional evidence demonstrates that nomophobia significantly predicts poor sleep quality via pre-sleep arousal pathways, with mediation analyses confirming that cognitive arousal is the primary mechanism linking smartphone dependency to sleep disturbance [43]. The progressive restriction protocol implemented here addressed withdrawal-related discomfort by incrementally extending restriction duration (30→60→120 min), mitigating the adherence barriers commonly observed with abrupt deprivation in nomophobic populations [24,44]. The convergence of objective actigraphic indices and subjective sleep quality improvements supports intervention efficacy, though actigraphy-subjective concordance demonstrates only moderate agreement in general populations and remains subject to measurement error from both modalities [32,33], with continuous wrist-worn monitoring offering ecologically valid assessment of sleep continuity across phases [32,33].

The significant reductions in pre-sleep anxiety (F(1,28) = 7.12, *p* = 0.012) and stress (F(1,28) = 6.45, *p* = 0.017) observed exclusively in the adapted group reflect the multicomponent intervention’s effects, integrating progressive restriction, psychoeducation, and pre-sleep relaxation. The specific contribution of breathing exercises versus psychoeducational content cannot be isolated, given the integrated design, rendering attribution of anxiety reductions primarily to autonomic mechanisms speculative [45]. Pre-sleep arousal encompasses cognitive hyperarousal (intrusive mentation, worry, rumination) and somatic arousal (physiological hyperactivation), both of which constitute perpetuating factors in contemporary models of insomnia [12]. The structured pre-sleep relaxation routine incorporating slow-paced breathing may have contributed through autonomic pathways [20,22], though psychoeducational normalization of smartphone separation anxiety likely conferred independent benefit. Meta-analytic evidence confirms that voluntary slow breathing increases vagally mediated heart rate variability during and following practice sessions [20], with theoretical mechanisms involving baroreceptor stimulation and brainstem autonomic regulation [21]. However, the present study did not measure heart rate variability or other autonomic indices, precluding direct verification of vagal modulation in our sample. Paced-breathing approaches demonstrate efficacy in addressing insomnia-relevant outcomes in controlled trials [22], providing an empirical rationale for their integration, though the specific autonomic contributions to observed sleep improvements remain unverified. Paced-breathing approaches demonstrate efficacy in addressing insomnia-relevant autonomic changes, providing an empirical rationale for integrating this technique into presleep routines for physically active populations [46].

The afternoon-specific cognitive performance improvements reflect circadian homeostatic interactions in individuals with an intermediate chronotype, assessed during typical academic schedules. Cognitive performance diurnal modulation shows strong chronotype dependence, with intermediate types exhibiting afternoon vulnerability during the post-lunch period (13:00–16:00 h), when homeostatic sleep pressure accumulates against declining circadian arousal [3,15]. Extreme morning and evening chronotypes demonstrate maximal impairment at different times, underscoring that afternoon vulnerability characterizes this specific population-schedule combination rather than representing universal cognitive nadir [47]. Sleep deprivation selectively impairs afternoon cognitive function through accumulated homeostatic sleep pressure and disrupted arousal maintenance [3]. The significant time × group × time-of-day interactions for psychomotor vigilance reaction time (F(1,28) = 7.48, *p* = 0.011) and choice reaction time (F(1,28) = 6.89, *p* = 0.014) indicate that improvements in sleep architecture restored cognitive capacity in the afternoon, when attentional vulnerability is typically greatest. The psychomotor vigilance task demonstrates established sensitivity to sleep-related variation in sustained attention and reaction speed [3,31]. Notably, reaction time improvements occurred without compromising response accuracy, arguing against speed-accuracy trade-offs and suggesting enhanced cognitive efficiency consistent with restorative sleep effects on prefrontal cortical function [2]. Meta-analytic evidence confirms dose-dependent effects of sleep restriction on vigilance and executive function [16], supporting the interpretation that even modest improvements in sleep quality can yield measurable cognitive benefits during circadian nadirs.

Physical performance outcomes remained stable across intervention conditions despite objective improvements in sleep. This absence of intervention-specific effects likely reflects multiple factors. First, the 14-day intervention duration may prove insufficient to influence neuromuscular adaptations in trained individuals, as explosive and maximal strength gains typically require 4–6 weeks of altered training-recovery cycles [26]. Second, the modest magnitude of sleep efficiency improvement (~5 percentage points) and onset latency reduction (~9 min), while clinically meaningful for cognitive restoration, may remain below the threshold necessary to detectably enhance maximal neuromuscular performance in young, physically active individuals already experiencing adequate baseline sleep (>400 min·night^−1^) [1,42]. Third, the evaluated tasks (vertical jumps, agility) assess ceiling-level explosive capacities in trained populations, demonstrating limited sensitivity to short-term sleep interventions when concurrent severe sleep restriction protocols are absent [26]. Significant time-of-day main effects confirmed expected circadian-driven afternoon performance peaks [17,18], validating assessment sensitivity while underscoring that intervention effects did not override established diurnal rhythms.

The differential sensitivity of cognitive versus physical performance to short-term sleep interventions reflects distinct neurobiological time courses. Cognitive restoration, mediated by prefrontal cortical recovery and adenosine clearance, responds rapidly to improved sleep continuity [2,16]. Conversely, neuromuscular performance adaptations depend on training load, recovery adequacy, and protein synthesis cycles operating over weeks rather than days [26]. Physical performance outcomes demonstrated expected diurnal effects with superior afternoon performance for squat jump (F(1,28) = 5.62, *p* = 0.025), countermovement jump (F(1,28) = 6.94, *p* = 0.013), and agility (F(1,28) = 7.21, *p* = 0.012), consistent with established late-afternoon peaks in neuromuscular and anaerobic performance [17,18]. The absence of intervention-specific changes suggests that short-term improvements in sleep initiation and efficiency, while sufficient to influence cognitive outcomes, prove insufficient to measurably enhance maximal or explosive physical performance in trained young adults over two weeks. Ceiling effects related to training status and test sensitivity may contribute to this null pattern, particularly given that adaptations in explosive performance typically require longer training-recovery cycles than the intervention duration permitted. These findings align with evidence indicating that improvements in physical performance require longer sleep durations or longer intervention periods in athletic populations [1].

The NMP-Q demonstrates acceptable psychometric properties (internal consistency, test–retest reliability, factorial structure) [27], though the construct of nomophobia validity as a distinct clinical entity remains debated [43]. Restricting enrollment to intermediate chronotypes [48] eliminated the confounding effects of extreme circadian preference. Blinding performance assessors minimized detection bias. A fixed test order was used, with standardized recovery intervals and clock-time-consistent procedural variability. Linear mixed-effects modeling accommodated repeated measures and inter-individual variability with appropriate statistical rigor.

Limitations warrant acknowledgment. The sample comprised physically active university students, potentially limiting generalizability to sedentary populations, older adults, or individuals with clinical insomnia. The 14-day intervention duration precludes conclusions regarding long-term efficacy and adherence sustainability. The absence of follow-up assessments precludes evaluation of sustained benefits post-intervention. Actigraphy, while validated for field-based assessment, lacks the polysomnographic precision required to quantify sleep architecture microstructure. Adherence to smartphone restriction and breathing protocols was monitored exclusively via self-reported daily logs, introducing potential reporting and social desirability biases. Objective verification tools, including application-based screen-time logs (e.g., iOS Screen Time, Digital Wellbeing) or automated device-use monitoring software, would strengthen adherence validity and permit dose–response analyses linking restriction magnitude to sleep outcomes [49]. The absence of objective smartphone-use data precludes verification of protocol compliance and limits mechanistic inferences about the temporal dynamics of adherence to the restriction. This represents a substantive limitation that may have attenuated observed effect sizes if adherence variability exceeded self-reported estimates. Future investigations should integrate objective monitoring technologies to precisely quantify adherence, differentiate compliant from non-compliant participants, and examine whether adherence trajectories moderate intervention efficacy [50]. The asymmetric intervention design, in which the adapted group received psychoeducation and relaxation components absent in the standard group, may introduce expectancy effects that influence subjective outcomes. Participants who are aware of receiving a ‘multicomponent’ intervention may report enhanced sleep quality and reduced anxiety, partially attributable to placebo-like mechanisms rather than to active intervention components [45]. However, significant improvements in objective actigraphic parameters (sleep efficiency, onset latency) alongside subjective ratings suggest that observed effects reflect genuine physiological changes rather than expectancy artifacts alone [32]. Cognitive performance enhancements, assessed via objective millisecond-precision computerized tasks, demonstrate minimal expectancy susceptibility, further supporting mechanistic validity [31]. Future trials should incorporate attention-matched control conditions (e.g., equivalent contact time with non-sleep-related psychoeducation) to isolate specific versus nonspecific intervention effects. Maintenance of stable sleep–wake schedules and training routines was monitored via participant self-report and daily logs without objective verification (e.g., training load quantification via accelerometry, heart rate monitoring, or validated training diaries). This reliance on behavioral compliance assumptions without external validation limits confidence in controlling for confounding variables, as undetected deviations in training volume or sleep timing may have influenced intervention effects independently of smartphone restriction protocols. Averaging sleep parameters across three nights provides stable phase estimates but does not capture adaptation trajectories to the intervention or account for high night-to-night variability, particularly during early intervention exposure, when behavioral adjustment processes remain dynamic [35]. Mechanistic inferences regarding pre-sleep arousal, autonomic regulation, and cognitive–emotional pathways remain tentative, as the study design did not include direct measures of mediators (e.g., pre-sleep cognitive arousal scales, heart rate variability indices, salivary cortisol trajectories). Observed improvements in sleep and cognitive outcomes are consistent with hypothesized mechanisms but do not definitively establish causal mediation pathways. Formal mediation analyses incorporating objective arousal biomarkers are required to validate proposed mechanistic frameworks [41]. Objective smartphone use verification was not implemented, limiting mechanistic inferences regarding dose–response relationships. Future research should integrate objective physiological markers (e.g., heart rate variability, cortisol) to elucidate psychophysiological pathways, examine dose–response relationships using factorial designs, investigate clinical populations with severe nomophobia, and conduct long-term follow-up to evaluate sustained effects and relapse prevention. Discussion interpretation acknowledges theoretical frameworks and external literature while recognizing that mechanistic, clinical, and applied claims extending beyond direct empirical observations remain tentative pending validation through mediator-focused designs, clinical population trials, and long-term follow-up investigations.

### Practical Recommendations

Findings from this preliminary randomized trial suggest that evening smartphone interventions may benefit from addressing smartphone-related anxiety and pre-sleep arousal beyond behavioral restriction alone [8]. Pending replication in larger samples with extended follow-up, practitioners working with physically active young adults experiencing moderate-to-high nomophobia might consider progressive restriction strategies (30→60→120 min) to potentially enhance adherence by attenuating withdrawal discomfort. Psychoeducational guidance grounded in cognitive–behavioral principles [12] should normalize transient anxiety during intentional disconnection and provide cognitive reframing strategies. Incorporating nightly slow-paced breathing protocols (5–6 breaths·min^−1^ for 10–12 min) offers an accessible, evidence-based strategy for enhancing vagal regulation and reducing pre-sleep arousal [20]. Practitioners may observe afternoon cognitive performance benefits that enhance motivation, particularly in populations that require sustained attention and decision-making capacity. Short-term sleep-focused smartphone interventions appear unlikely to directly improve maximal or explosive physical performance in trained young adults beyond normal diurnal variation [17,18]. These findings support integrating structured smartphone management and pre-sleep regulation strategies into recovery education programs for physically active students and in athlete development settings.

## 5. Conclusions

This randomized controlled trial demonstrates that adapted smartphone interventions combining progressive restriction, psychoeducational guidance, and pre-sleep relaxation produce statistically significant and theoretically meaningful improvements in sleep efficiency, sleep onset latency, pre-sleep psychological arousal, and afternoon cognitive performance in physically active university students with moderate-to-high nomophobia. Generalizability to broader populations, clinical significance in treatment-seeking samples, and durability beyond the 14-day intervention period remain empirically unresolved and require independent replication with larger samples, extended follow-up, and diverse demographic cohorts. The findings support a mechanistic framework wherein cognitive–behavioral strategies targeting anticipatory anxiety and autonomic regulation address core pathways linking smartphone dependency to sleep disturbance. The afternoon-specific cognitive enhancements underscore the functional significance of improvements in sleep architecture. These results provide preliminary empirical support for multicomponent, mechanism-informed behavioral interventions addressing technology-related sleep disturbances in physically active young adults with elevated nomophobia. Comparative efficacy trials incorporating clinical populations and pharmacological comparator arms are required to evaluate relative treatment benefits and establish clinical positioning.

## Figures and Tables

**Table 1:** Baseline Anthropometric and Psychological Characteristics of Participants Randomized to Standard Smartphone Restriction and Adapted Smartphone Intervention Groups.

Variable	Standard Restriction Group (*n* = 15)	Adapted Intervention Group (*n* = 15)	*p*-Value
Age (years)	21.9 ± 1.23	21.86 ± 1.20	0.886
Height (cm)	175.94 ± 4.01	175.70 ± 4.26	0.883
Body mass (kg)	70.68 ± 2.82	71.02 ± 3.52	0.784
Sex (men/women)	10/5	9/6	0.720
NMP-Q score	82.80 ± 5.80	84.93 ± 9.20	0.453

Values are presented as mean ± standard deviation. Between-group comparisons were conducted using independent-samples *t*-tests. NMP-Q = Nomophobia Questionnaire. Baseline NMP-Q scores ranged from 68 to 103 across the sample (moderate severity: *n* = 24; high severity: *n* = 6). No statistically significant differences were observed between groups at baseline (all *p* > 0.05), confirming successful randomization.

**Table 2:** Baseline Descriptive Statistics and Between-Group Comparisons for Primary Outcome Variables.

Outcome Variable	Standard Restriction (*n* = 15)	Adapted Intervention (*n* = 15)	*p*-Value
**Sleep Parameters**			
Sleep efficiency (%)	82.1 ± 2.3	85.2 ± 2.1	0.29
Sleep onset latency (min)	30.3 ± 5.8	27.1 ± 5.2	0.14
Total sleep time (min)	403.9 ± 13.4	411.2 ± 12.9	0.17
Subjective Sleep Quality (VAS)	5.8 ± 1.1	6.1 ± 1.2	0.48
Pre-Sleep Anxiety (VAS)	5.2 ± 0.9	5.4 ± 1.0	0.58
Pre-Sleep Stress (VAS)	5.1 ± 0.8	5.3 ± 0.9	0.52
**Cognitive Performance (Morning)**			
PVT reaction time [20]	454.2 ± 14.6	456.8 ± 15.2	0.64
CRT reaction time [20]	398.7 ± 12.8	401.3 ± 13.1	0.6

Values are mean ± standard deviation. Between-group comparisons were conducted via independent-samples *t*-tests. VAS = Visual Analog Scale (0–10); PVT = Psychomotor Vigilance Task; CRT = Choice Reaction Time. No statistically significant baseline differences observed (all *p* > 0.05).

**Table 3:** Effects of Standard Smartphone Restriction and Adapted Smartphone Intervention on Objective Sleep Parameters.

Variable	Adapted Intervention Group (*n* = 15)	Standard Restriction Group (*n* = 15)	Time × Group Interaction
	Baseline	Post-Intervention	Baseline
Sleep Efficiency (%)	85.10 ± 2.30	90.60 ± 2.60 **	82.50 ± 2.10
Sleep Onset Latency (min)	27.40 ± 5.30	18.20 ± 4.90 *	30.10 ± 5.60
Total Sleep Time (min)	410.60 ± 13.20	424.10 ± 14.10	404.80 ± 12.90
Wake After Sleep Onset (min)	58.40 ± 7.80	49.10 ± 7.10	56.20 ± 6.90

Values are presented as mean ± standard deviation. Bonferroni-adjusted post hoc comparisons: * *p* < 0.05, ** *p* < 0.01 relative to baseline within group.

**Table 4:** Effects of Time of Day and Intervention on Cognitive Performance in Standard Smartphone Restriction and Adapted Smartphone Intervention Groups.

Variable	Adapted Intervention Group (*n* = 15)				Standard Restriction Group (*n* = 15)			
	Baseline Morning	Post-Intervention Morning	Baseline Afternoon	Post-Intervention Afternoon	Baseline Morning	Post-Intervention Morning	Baseline Afternoon	Post-Intervention Afternoon
PVT RT [20]	455.00 ± 14.00	431.00 ± 13.00	439.00 ± 15.00	397.00 ± 14.00 **	456.00 ± 15.00	454.00 ± 14.00	446.00 ± 14.00	445.00 ± 15.00
PVT Lapses (*n*)	7.30 ± 0.80	5.80 ± 0.80	6.30 ± 0.90	3.40 ± 0.90 *	7.50 ± 0.90	7.10 ± 0.80	6.80 ± 0.80	6.10 ± 0.90
CRT [20]	404.00 ± 12.00	389.00 ± 11.00	390.00 ± 12.00	358.00 ± 11.00 **	402.00 ± 13.00	399.00 ± 12.00	388.00 ± 12.00	386.00 ± 12.00
CRT Accuracy (%)	95.60 ± 2.10	96.10 ± 2.00	95.80 ± 2.30	96.40 ± 2.10	95.40 ± 2.00	95.20 ± 2.10	95.70 ± 2.20	95.50 ± 2.30

Values are presented as mean ± standard deviation. Morning assessments conducted at 09:00 h; afternoon assessments at identical clock time across sessions; PVT = Psychomotor Vigilance Task; RT = reaction time; Lapses = vigilance lapses (RT ≥ 500 ms); CRT = Choice Reaction Time. Bonferroni-adjusted post hoc comparisons: * *p* < 0.05, ** *p* < 0.01 relative to baseline within time-of-day condition.

**Table 5:** Effects of Time of Day and Intervention on Physical Performance in Standard Smartphone Restriction and Adapted Smartphone Intervention Groups.

Variable	Adapted Intervention Group (*n* = 15)				Standard Restriction Group (*n* = 15)			
	Baseline Morning	Post-Intervention Morning	Baseline Afternoon	Post-Intervention Afternoon	Baseline Morning	Post-Intervention Morning	Baseline Afternoon	Post-Intervention Afternoon
Agility (s)	6.54 ± 0.20	6.51 ± 0.20	6.45 ± 0.20	6.46 ± 0.21	6.50 ± 0.15	6.49 ± 0.16	6.42 ± 0.15	6.42 ± 0.16
CMJ (cm)	38.30 ± 1.90	38.40 ± 1.90	39.70 ± 2.00	39.90 ± 2.00	39.00 ± 2.00	39.00 ± 2.00	40.20 ± 2.10	40.10 ± 2.10
SJ (cm)	35.80 ± 1.60	36.00 ± 1.60	36.90 ± 1.70	37.10 ± 1.70	35.40 ± 1.70	35.50 ± 1.70	36.60 ± 1.80	36.70 ± 1.80

Values are presented as mean ± standard deviation; Morning assessments conducted at 09:00 h; afternoon assessments at identical clock time across sessions; CMJ = Countermovement Jump; SJ = Squat Jump. No significant time × group or time × group × time-of-day interactions emerged (all *p* > 0.05). Significant main effects of time of day were observed for all variables (all *p* < 0.025), with superior afternoon performance.

## Data Availability

The data supporting the findings of this study are not publicly available due to privacy and ethical restrictions, as they contain sensitive information about study participants. However, the data are available from the corresponding author upon reasonable request, provided that permission is obtained from the relevant ethics committee.
